# Heat exposure in pregnancy: a silent risk for neural tube defects in Pakistan

**DOI:** 10.1097/MS9.0000000000003802

**Published:** 2025-08-28

**Authors:** Hassan E. Muhammad, Asad Ali Ahmed Cheema

**Affiliations:** a Department of Medicine, Gujranwala Medical College, Gujranwala, Pakistan; b Department of Medicine, International School of Medicine, International University Kyrgyzstan, Bishkek, Kyrgyzstan

*To the Editor,*

Neural tube defects (NTDs), including anencephaly and spina bifida, are among the most common congenital anomalies globally, contributing to lifelong disability, increased healthcare costs, and substantial perinatal mortality. An estimated 214 000 to 322 000 pregnancies are affected annually worldwide^[1]^. This manuscript complies with the TITAN 2025 guideline for transparency in the use of AI in scholarly publishing^[2]^.

In low- and middle-income countries (LMICs) such as Pakistan, the actual burden of NTDs remains underestimated due to a lack of national birth defect surveillance. However, regional studies report prevalence rates of 12–14 per 1000 live births^[3,4]^, corresponding to 38.6–124.1 per 10 000 live births, which is substantially higher than those in high-income countries^[5]^.

While folic acid supplementation is well-established as a preventive measure, emerging evidence implicates maternal hyperthermia during early gestation from febrile illness, environmental heat exposure, or hot water immersion as a teratogenic risk factor. A landmark meta-analysis by Moretti *et al* (2005) found a significant association between maternal hyperthermia and the risk of NTDs^[6]^. Additionally, a 2024 population-based case-control study in Georgia, USA, by LaPointe *et al* reported dose-dependent increases in NTD risk with exposure to 1–2, 3–5, and 6+ consecutive days of extreme ambient heat during the periconception period, showing adjusted odds ratios of 1.09, 1.18, and 1.29, respectively^[7]^.

Despite this compelling data, public awareness in Pakistan remains low. Cultural practices, such as taking hot baths during early pregnancy, are often standard and unchallenged due to a lack of risk-communication initiatives. Moreover, primary care and antenatal providers, including community health workers, are rarely equipped to advise women on thermal safety during critical periods of neural development.

To address this gap, we urge the following actions: (1) incorporate maternal heat-risk education into national antenatal care guidelines; (2) train community health workers and lady health visitors to counsel pregnant women on avoiding excessive heat exposure; and (3) establish a congenital anomalies registry in Pakistan to quantify the burden, monitor trends, and guide interventions.

In conclusion, while folic acid supplementation remains essential, the teratogenic risk of maternal hyperthermia is an under-recognized yet preventable factor in Pakistan. Urgent integration of thermal safety education into maternal health services is vital to reducing the national burden of neural tube defects.

## Data Availability

Not applicable – No new data were generated or analyzed in this letter to the editor.

## Peer and provenance statement

Not commissioned; externally peer reviewed.
